# The Tarsal Bone Test: A Basic Test of Health Sciences Students' Knowledge of Lower Limb Anatomy

**DOI:** 10.1155/2014/939163

**Published:** 2014-07-08

**Authors:** José Manuel Castillo-López, Juan Antonio Díaz-Mancha, Alberto Marcos Heredia-Rizo, Lourdes María Fernández-Seguín, Juan Polo-Padillo, Gabriel Domínguez-Maldonado, Pedro V. Munuera

**Affiliations:** ^1^Department of Podiatry, University of Seville, Facultad de Enfermería, Fisioterapia y Podología, Calle Avicena S/N, 41009 Sevilla, Spain; ^2^Department of Physiotherapy, University of Seville, Facultad de Enfermería, Fisioterapia y Podología, Calle Avicena S/N, 41009 Sevilla, Spain; ^3^Department of Preventive Medicine and Public Health, University of Seville, Facultad de Medicina, Avenida Sánchez Pizjuán S/N, 41009 Sevilla, Spain

## Abstract

*Objectives.* The aim of the present study was to design an easy-to-use tool, the tarsal bone test (TBT), to provide a snapshot of podiatry students' basic anatomical knowledge of the bones of the lower limb. *Methods.* The study included 254 podiatry students from three different universities, 145 of them were first-year students and 109 were in their fourth and final years. The TBT was administered without prior notice to the participants and was to be completed in 5 minutes. *Results.* The results show that 97.2% of the subjects (*n* = 247) correctly labelled all tarsal bones, while the other 2.8% (*n* = 7) incorrectly labelled at least one bone, that was either the cuboid (7 times) or the navicular (6 times). Although only one fourth-year student inaccurately identified one bone, no significant differences in the distribution of the correct and incorrect responses were found between first and fourth-year students. *Conclusions.* The TBT seems to be a straightforward and easy-to-apply instrument, and provides an objective view of the level of knowledge acquired at different stages of podiatry studies.

## 1. Introduction

A profound knowledge of human anatomy has been and continues to be an essential, cross-discipline, and structuring component of the academic curriculum of various fields of the health sciences [1]. Medical students as well as different specialists consider gross anatomy, physiology, and pharmacology to be the three basic disciplines for preparation for daily clinical activity [2–4]. The development and implementation of the European Higher Education Area [5] have been linked to the adoption of a new educational model, which has even been termed as “paradigm shift.” As a result, in health sciences, the basic science subjects, including anatomy, need to be integrated with the clinical skills that the students will require in the development of their future career.

In higher education, there have been major modifications made to Health Sciences degree courses in order to harmonize educational systems Europe wide [6]. Spain universities have been engaged in a process of profound change in their educational structures since the 1990s. These changes have affected universities' academic structures, undergraduate and graduate syllabi, length in years of the courses, and classroom teaching methods and learning activities. Consequently, the traditional teaching techniques of anatomy, such as dissection by students, prosection and demonstrations, living and surface anatomy, theoretical lectures, and the use of models (e.g., plastinated material), are giving way to such new learning techniques as problem-based learning, computer-assisted learning, virtual anatomy, and virtual dissection techniques which are incorporated into seminars, small groups, personal academic tutorials, and specific project-based tasks (“learning by doing”) [7].

Recent research seems to show that medical students have a knowledge level of the gross anatomy of the musculoskeletal system that is sparse in comparison with students of other health professions [8–10]. This, coupled with the need to evaluate possible changes in how knowledge is acquired in the context of new teaching methods, has motivated several studies in this area. To this end, various instruments of measurement have been designed. One such is the carpal bone test [11], in which the participant must label properly the carpal bones on a picture of the bones of the wrist and hand. The bones of the carpal region were chosen as a benchmark of anatomical knowledge since they were easily and objectively examined and had musculoskeletal clinical relevance in a number of disciplines. This test has been used to evaluate the level of knowledge of students in different health science disciplines [10, 12]. The carpal bone test appears to be a valid instrument with which we assess knowledge of anatomy for health care professionals who provide care for musculoskeletal conditions of the upper limb. However, in the opinion of the authors, it could be beneficial to increase the battery of this type of tests with the inclusion of other parts of the body. There is an apparent gap in medical science students' learning of knowledge related to specific anatomical regions that needs to be addressed [13]. Of different anatomical regions, the foot and ankle have been observed to be those for which students show least confidence in regard to clinical diagnoses and physical examination [13]. In addition, musculoskeletal pain conditions of the foot and ankle are becoming ever commoner and already represent a major challenge and economic burden for public health services [14], since they affect around 20% of the middle-aged to older population (over 45 years old) [15]. Because the large bones of the lower limb are easily recognizable (femur, tibia, and fibula), we think that the tarsal bones are the best suited to evaluate the knowledge of anatomy. Moreover, knowledge of tarsal bone anatomy would also seem to be relevant for health professions directly concerned with foot care.

The objective of the present study was therefore to design a tarsal bone test, and, using that test, to provide a snapshot of Spanish podiatry students' basic anatomical knowledge of the bones of the lower limb.

## 2. Materials and Methods

The study was conducted during the last two weeks of May 2013 among podiatry undergraduates in the Spanish Universities of Extremadura (102 students), Malaga (78 students), and Seville (74 students) during their 2012-2013 academic year. The University of Seville has a history of over 500 years, including a long tradition in the field of health and social care sciences. It is Spain's third university in terms of student numbers. Together with the University of Malaga, founded in 1972 and now with over 40 000 students, it sets up the “Andalucía TECH” educational project which, in 2010, attained the category of Campus of International Excellence awarded by Spain's Ministry of Education. The University of Extremadura was founded in 1973 and had approximately 25 000 students in the 2012-2013 academic year.

In Spain, podiatry became a university discipline in 1988, beginning as a three-year degree course. In 2009, it became a four-year course, with a total of 240 European Credit Transfer System (ECTS) in four academic years. The first graduates of this latter course received their degrees in June 2013. Their academic training includes specific anatomical content only during the first academic year, as also in other health disciplines in Spain. Since they are training to be specialists in foot and lower limb care, the authors decided to evaluate the tarsal bone test on this specific group of students. Knowledge of foot and lower limb anatomy is worked on transversally across different subjects that the students take in their four undergraduate years.

In the three universities to which the study's participants belonged, two 6-ECTS-credit subjects of anatomy are taught, each during the first academic year. The first deals with content of general human anatomy and the second with specific anatomy of the lower limb. In the University of Seville, these two subjects have total of 95 hours of theory classes and 25 hours of practical classes in the dissecting room. In the University of Malaga, the corresponding figures are 70 hours of theory and 50 hours of practical content (only 16 in the dissecting room), and, in the University of Extremadura, they are 92 hours of theory and 28 hours of practical content (only 4 hours in the dissecting room).

**Figure 1:**
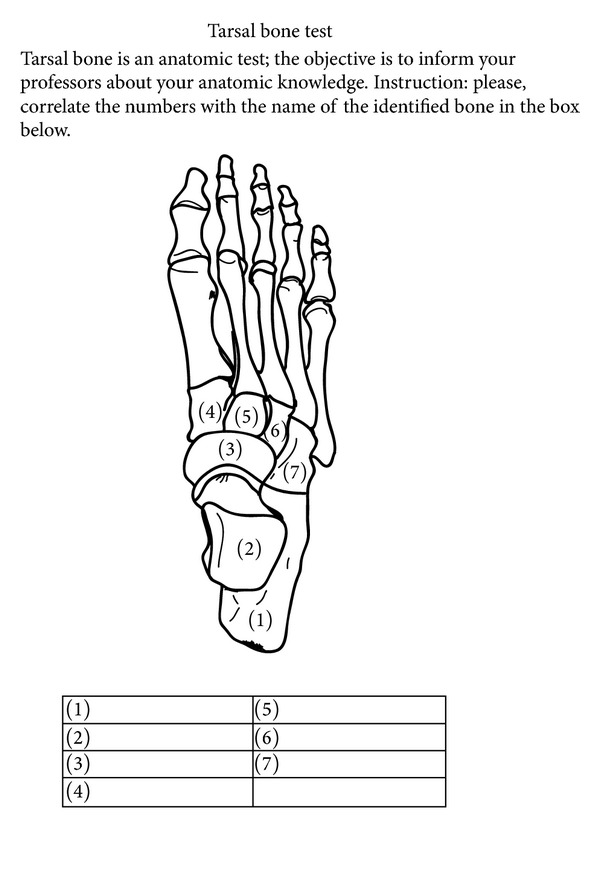
Tarsal bone test form.

Two hundred and fifty-four (*n* = 254) tarsal bone test questionnaires were completed by first-year (145 questionnaires) and final-year (109 questionnaires) podiatry students in the presence of one of the authors. The test consists of a drawing of the bones of the foot, with lines and numbers to label the name of each tarsal bone (Figure 1). It was administered without prior notice to the student volunteers, to obviate their preparing of the answers in advance. The researcher briefly explained that the test was to be completed in a maximum time of 5 minutes and that they were allowed not to speak to each other during that time. The tests were handed in to the researcher as they were completed. Students who completed the tests were encouraged to remain silent so as not to disturb the others or provide them hints for the answers. The study was approved by the institutional research ethics committee. The participants signed an informed consent form prior to their inclusion in the study.

### 2.1. Statistics

The data were analysed with the SPSS for Windows (SPSS Science, Chicago, IL, USA) statistical software package. The results will be presented using descriptive statistics. Differences in the distribution of the responses between groups were analysed with the *χ* ^2^ test, using Fisher's exact test when the condition was not satisfied that the expected values of at least 80% of the cells in the contingency table were greater than 5. The statistical analysis was conducted at a 95% confidence level, with *P* < 0.05 considered as statistically significant.

## 3. Results and Discussion

Of the 261 students who offered to participate, 254 handed in the completed questionnaire to the corresponding researcher in the classroom. The other 7 refused to form part of the study.  Table 1 presents the data on the distribution by gender, age, and academic year of the participants.

Of the 254 participants, 97.2% (*n* = 247) correctly labeled the tarsal bones, while the other 2.8% (*n* = 7) incorrectly labeled at least one. No questionnaire had any response left blank. The incorrectly labeled bones were the cuboid (7 times) and the navicular (6 times). In particular, one fourth-year student wrongly labeled just the cuboid. The other 6 were first-year students who labeled wrongly the cuboid and/or the navicular (Table 2).

Although only one fourth-year student made a mistake, and the rest were first-year, Fisher's exact test showed no significant differences in the distribution of the correct and incorrect responses between the two groups, whether for the navicular (*P* = 0.262) or for the cuboid (*P* = 0.244).

With respect to the distribution by university, all of the University of Malaga students labeled the bones correctly. One University of Seville (first-year) student incorrectly labeled the cuboid and the navicular. At the University of Extremadura, one fourth-year and three first-year students incorrectly labeled the cuboid, and two first-year students confused the cuboid with the navicular. While the *χ* ^2^ test showed the differences between universities not to be statistically significant in the case of the navicular (*P* = 0.481), they were so in the case of the cuboid (*P* = 0.042).

To the best of the authors' knowledge, this is the first transversal study to have been conducted at different universities to investigate podiatry undergraduates' level of knowledge of lower limb anatomy using the tarsal bone test. The results indicate that the students evaluated had a high degree of knowledge and retentive capacity of the names of the tarsal bones, with only locating the cuboid and navicular bones presenting any difficulty.

Previous studies have explored the instructional techniques used to teach anatomy and evaluated the anatomical knowledge of different medical speciality professionals [4], of course directors of medical schools in the USA [16] and students of physiotherapy [17–19], nursing [18, 20], and medicine [7, 19]. For this, use has sometimes been made of a test very similar to that used in the present study. This is the carpal bone test [10–12], in which students are presented with a drawing of the carpal bones which they should be able to label correctly.

While the present instrument might be seen as rather too rough-and-ready tool with which we can measure knowledge of anatomy, it is straightforward and easy to apply and provides an objective view of the level of knowledge acquired at different stages of podiatry studies. The three participating universities have great national prestige regarding their training in podiatry. Anatomy is taught in the first year by specialized teachers belonging to specific anatomy departments (Department of Anatomy and Forensic Medicine at the University of Malaga; Department of Anatomy, Cell Biology and Zoology at the University of Extremadura; and Department of Anatomy and Embryology at the University of Seville).

The test was given at the end of the first and fourth years of the undergraduate degree course in podiatry. At that point, the first-year students have already completed all the hours of anatomy they will be taught in their course. Over the following years, this knowledge is reinforced by the use made of it in the theoretical and practical classes of the other subjects they have to take. Most of the mistakes made corresponded to first-year students, suggesting that any gaps in anatomical knowledge are filled during the following three years in their course. By the time the fourth-year students come to the end of their classes and are about to become professionals with the full capacity to practice as podiatrists, they must possess an appropriate level of knowledge of anatomy, among other topics. As noted above, anatomy is nothing other than an essential basic discipline for their subsequent clinical reasoning as health professionals. Indeed, health care professionals need a profound and sound knowledge of anatomy and human function [21]. In addition, anatomical knowledge has been considered as “fundamental” or very “relevant” for such everyday practices as imaging diagnosis, physical examination, and performing therapeutic procedures, among others [4]. This could be one of the main reasons for the high complete success rate (97.2%) that was observed on the tarsal bone test, so that the authors would suggest taking these results as a “gold standard” against which we compare the results of future research that might use this test. We found that 3 of the 7 students who labeled some bone incorrectly confused the navicular with the cuboid. Although they are of structures clearly distinct in shape and location, the authors think the confusion might be because the common name used in podiatry in Spain for the navicular is “escafoides” which resembles the Spanish word for the other bone “cuboides.” Also, of course, they are anatomically contiguous. Another reason for this confusion may be that, in memorizing their names, the students do so in some particular order (as some of them stated to us), and this order did not match the numbers that appeared in the test.

Although this aspect of confusion was limited to a very small number of students, it needs to be taken into account given the clinical importance of the two in the biomechanics of the midfoot. The so-called cuboid syndrome, for instance, is a relatively common and painful condition of the lateral midfoot that accounts for almost 20% of professional ballet dancers foot and/or ankle injuries [22] and has also been related to subjects with plantar flexion/inversion ankle sprains [23]. In addition, the tarsal navicular bone is not only a common area for stress injuries, but it also often presents poor subsequent healing due to the unusual blood supply that it requires [24].

Among the three participating universities, the University of Extremadura is the one which employs fewest hours of study in anatomy in the dissecting room. Except for one student at the University of Seville, the rest of the students who made mistakes were at that university. It was not an objective of this study to examine the relationship between the number of hours spent studying the anatomy in the dissecting room and the level of anatomical knowledge attained, but this finding would be consistent with those authors who argue that dissection is a cornerstone for the proper teaching of anatomy [1, 7, 25]. Dissection is a common method of instruction for anatomy in several health care disciplines, although predictions of the future of the teaching of these disciplines indicate that the time spent on dissection will decrease as more time is allocated to imaging and palpation techniques [19]. Moore argues that the sensation of touch between physician and patient is essential [26] and that this is best learnt early on in the dissecting room. Chirculescu et al. [7] claim, however, that one prejudice which should be rejected is that teaching and learning anatomy through virtual reality are enemies which aim to substitute real dissection and instead propose that it should be viewed as a useful method complementing approaches to anatomy through the cadaver. In addition, previous studies on the level of factual knowledge in students studying podiatric medicine report no differences between those who had followed a novel learning approach, such as problem-based learning, and those who had followed a traditional teaching approach [27]. Although it remains controversial, it would seem that, while a more student-centered approach, based on active learning, independent study, and the resolution of cases and/or problems, may be more effective in developing professional competencies especially in the social and cognitive domains, it is not directly related to the acquisition of basic knowledge [28].

Anatomical competence is critical for future professionals in any of the health science disciplines, not only podiatry. According to older [25], the more a physician remembers anatomical facts, the better his or her clinical skills will be. Indeed, this is so much so that Cahill et al. [29] have expressed concern that of the 80 000 avoidable deaths per year in the USA at least some can be attributed to anatomical incompetence, although a recent study found only a weak to moderate correlation between the marks in anatomy and in clinical skills obtained by 538 medical students [30].

## 4. Conclusions

The tarsal bone test was developed and tested on first- and final-year podiatry students to monitor the quality of the teaching they had received and to evaluate their retention of knowledge of lower limb anatomy. The results were that 97.2% of the tests were completed correctly, with the cuboid and navicular bones accounting for all the mislabeled cases. The authors propose that these results might serve as a “gold standard” for comparison with those obtained in other studies in which this test is used.

## Figures and Tables

**Table 1:** Characteristics of the study participants.

	Total sample	University of Extremadura		University of Seville		University of Malaga	
		First year	Fourth year	First year	Fourth year	First year	Fourth year
*N*	254	64	39	34	39	47	31
Age (years)	22.29 ± 4.15	20.89 ± 2.88	24.28 ± 3.45	19.71 ± 2.42	23.64 ± 3.30	21.72 ± 5.75	24.84 ± 3.86
Gender (male/female)	72/182	18/46	11/28	3/31	15/24	22/25	3/28

**Table 2:** Distribution of the bones labeled incorrectly.

	University	Academic year	Mislabeled bones
Student 1	Seville	First	Cuboid navicular
Student 2	Extremadura	Fourth	Cuboid
Student 3	Extremadura	First	Cuboid
Student 4	Extremadura	First	Cuboid
Student 5	Extremadura	First	Cuboid
Student 6	Extremadura	First	Cuboid navicular
Student 7	Extremadura	First	Cuboid navicular

## Limitations

In considering possible limitations of this study, it must be borne in mind that the extreme simplicity of the tarsal bone test means that it provides only a minimal insight into the students' anatomical knowledge. For a more exhaustive evaluation of health care students' knowledge retention, a more extensive database of human anatomy should be used, together with the inclusion of elements from other disciplines (radiology, neuroanatomy, etc.). The authors' intention in this preliminary study was to develop a new, objective, and easily administered test with the goal of evaluating anatomical knowledge of the lower limb. As suggested above, researchers in this area should include students from different universities and in different years of the curriculum [10]. Since the present results correspond to a particular area in southern Spain, any generalization to the overall population of podiatry students would have to be made with caution.
